# Retrospective assessment of metabolic syndrome components in early adult life on vegetarian dietary status

**DOI:** 10.3389/fpubh.2022.945805

**Published:** 2022-08-16

**Authors:** Rachita Pandya, Rashed Abdelaal, Joe W. Chen, Shabana Masood, Zohray Talib, Hani Atamna, Mohsin Yakub, Sherif S. Hassan

**Affiliations:** ^1^California University of Science and Medicine, Colton, CA, United States; ^2^Claremont Graduate University, School of Community and Global Health, Claremont, CA, United States; ^3^Cairo University, Cairo, Egypt

**Keywords:** metabolic syndrome, vegetarian, young adults, retrospective analysis, propensity scoring

## Abstract

**Background:**

Metabolic syndrome (MetS) encompasses several clinical presentations that include truncal obesity and insulin resistance at its core. MetS afflicts 23% of the adult US population, increasing their risk of diabetes and cardiovascular disease. Many studies have indicated the importance of a vegetarian diet in improving overall health and more specifically MetS components. Unfortunately, these findings have been inconsistent and cannot be extended to examine effects on MetS incidence in the younger adult population.

**Objective:**

This study aimed to conduct a retrospective analysis of a vegetarian vs. non-vegetarian dietary status in young adults (age 18–24) based on MetS components in later adulthood (age 20–30). This study focuses on elucidating any relationship between a vegetarian diet and MetS components of central obesity, hypertension, and hyperlipidemia. Methods: Waves 3 and 4 data were acquired from AddHealth. One-to-one propensity score matched vegetarians to non-vegetarians in a cohort of 535 women and 159 men. Logistical regression assessed the relationship between vegetarian status and MetS components, including truncal obesity (cm), hypertension (normal, pre-HT, HT1, and HT2), and hyperlipidemia (high and low). Results MetS components from ages 20 to 30 are not associated with vegetarian dietary status. Truncal obesity [*N* = 694; *M* = 92.82 cm; OR 0.999; *p* = 0.893; 95% CI (0.980, 1.017)]; hypertension [*N* = 694; OR 0.949; *p* = 0.638; 95% CI (0.764, 1.179)]; hyperlipidemia [*N* = 694; OR 0.840; *p* = 0.581; 95% CI (0.453, 1.559)].

**Conclusion:**

Current study results were consistent with previous findings suggesting that consumption of a vegetarian diet cannot be directly linked to MetS outcomes. However, further investigation should be completed as MetS is a risk factor for several chronic diseases.

## Introduction

Metabolic syndrome (MetS) is defined as “a cluster of metabolic disorders” by the American Heart Association (1). Approximately 23% of adults in the United States are affected by MetS, increasing the risk for diabetes mellitus, stroke, and cardiovascular disease, along with increased atherosclerosis (1). Being overweight or obese, along with the lack of physical activity, genetic factors, and aging contribute to MetS (2). According to the International Diabetes Federation (IDF), central obesity is a prerequisite for MetS diagnosis and is independently associated with insulin resistance and other MetS components (3). In addition to central obesity, the IDF definition also requires at least two of the following four factors: elevated blood pressure (systolic blood pressure (BP) ≥130 or diastolic BP ≥85 mm Hg), reduced High-density lipoprotein (HDL) cholesterol in men (<40 mg/dL or 1.03 mmol/L) and in women (<50 mg/dL or 1.29 mmol/L), elevated triglycerides (≥150 mg/dL or 1.7 mmol/L), and elevated fasting plasma glucose (FPG) [≥100 mg/dL or 5.6 mmol/L], or previously diagnosed type 2 diabetes (if FPG exceeds 100 mg/dL or 5.6 mmol/L) (3).

Metabolic syndrome risk mitigation includes weight loss and increased physical activity. Consuming more fruits, grains, fish, and vegetables has also been shown to decrease the risk of MetS, and are key components of a vegetarian diet, based on a review on vegetarian diets published through the American Dietetic Association (4). Other studies have also stated equivalent results. In a cross-sectional analysis of 773 subjects who were randomly selected from the Adventist Health Study 2 and whose dietary pattern was assessed with a quantitative, self-administered food frequency questionnaire, a vegetarian diet was associated with a significantly lower risk of MetS and a greater favorable profile of metabolic risk factors after adjusting for demographics and lifestyle factors (5). A separate, evidence-based review showed that vegetarian diets were linked to decreased mortality from ischemic heart disease (IHD) (6). Compared to non-vegetarians, decreased blood pressure, decreased low-density lipoprotein (LDL) cholesterol levels, and lower rates of type 2 diabetes and hypertension were present in vegetarians (4). The same study found that vegetarians had an overall decreased incidence of cancer and lower body mass index (4). A case-control study looked at patients from eight Indian hospitals and found that consuming 3 or more servings of green leafy vegetables per week was linked to a significantly decreased risk of heart disease (7). The study consisted of 350 cases of acute myocardial infarction participants and 700 controls matched by age and sex (7). Prior studies also show that increased duration of a vegetarian diet resulted in better health outcomes. In a pooled analysis of cohort studies with participants younger than 90 years old, vegetarians had a 24% decrease in mortality from IHD compared to non-vegetarians. Decreased mortality from IHD was noted in the vegetarian group for individuals <65 years old, specifically for those who followed their dietary pattern for at least 5 years (8). Another study analyzed the impact of a long-term vegetarian diet on biochemical markers of cardiovascular disease risk and antioxidants through a community health project. The treatment group consisted of 30 vegetarians with a mean age of 44.2 ± 9 years and maintenance of a vegetarian diet for a mean of 21.8 ± 12.2 years. The age-matched control group consisted of individuals who were not following a vegetarian diet. The results of that study indicated that participants who followed long-term vegetarianism had a lower coronary heart disease risk profile and a more favorable antioxidant status than healthy omnivores (9).

Long-term vegetarianism also had positive outcomes for diabetics. In a study among 652 patients with diabetes, 39% of patients treated with insulin and 71% of patients treated with oral hypoglycemic agents were able to discontinue medication use after following a low fat (10% energy) vegetarian diet. The low-fat vegetarian diet also resulted in a decrease in fasting blood glucose by 24%, serum cholesterol by more than 20%, and triacylglycerol by more than 30% (10). The Seventh-Day Adventist Study began with a cohort of 25,698 adults in 1960 and followed them for 21 years showing a decreased incidence of diabetes based on participants' self-report in vegetarians than in non-vegetarians (11).

Although many studies exist on the long-term health effects of a vegetarian diet, there are no studies that retrospectively assessed central obesity, hypertension, and hyperlipidemia in relation to vegetarian status in young adults. Retrospectively assessing their dietary status based on metabolic health outcomes in later years would allow us to understand the effects of a vegetarian diet on health outcomes in younger adults (12).

A 2015 study by Chiu et al. focused on cross-sectional and longitudinal comparisons of metabolic profiles between vegetarians and non-vegetarians (13). While their study found that vegetarian diets had a significant benefit on metabolic traits, their study design presented a key limitation: participants were re-evaluated at unequal time points, with many of them having only a single visit. In contrast to these findings, a 2019 systematic review and meta-analysis of cross-sectional studies conducted by Picasso et al. suggested that a vegetarian diet is not statistically associated with a lower risk of MetS when compared with an omnivorous diet (14). However, much heterogeneity among the studies was present, and many of the studies did not adjust for confounding variables. Together, these issues illuminate the need for further investigation into the relationship between MetS and vegetarian diets using a more robust methodology.

The aim of this study was to retrospectively examine the dietary status in young adults (18–24; Wave III) and its relation to MetS components, namely hypertension, central obesity, and hyperlipidemia in 24–32-year-old participants (Wave IV). The secondary longitudinal data from the AddHealth study were employed to retrospectively compare vegetarian status based on MetS components, adjusting for race/ethnicity, socioeconomic status (SES), and gender as covariates. The AddHealth Study is an ongoing study that began in 1994 (Wave I), with the original goal of expanding the understanding of adolescent health and behavior. Since the study only gathered limited data (namely height and weight) in its first two waves, our study looked at the Wave III data in which participants were 18–24 years old. This age also typically coincides with completing high school and the newfound independence that follows (15). We hypothesized that following a vegetarian diet in young adulthood vs. a non-vegetarian diet would be associated with a lower prevalence of MetS components in later years of adulthood, specifically hypertension, central obesity, and hyperlipidemia.

## Methods

### Data source

The National Longitudinal Study of Adolescent Health (Add Health) was a longitudinal study based on a clustered sampling design of a sample of grades 7–12 in the United States from 1994 to 1995. Systematic sampling and implicit stratification were used to make sure that the selected 80 high schools were representative of U.S. schools in regard to ethnicity, region of country, urbanicity, type, and size. Also, it was ensured that selection probability was proportional to the school size. The individuals in the study were followed with four in-home interviews through adolescence and transition to adulthood. These interviews were divided into Waves (Wave I [1994–95]; Wave II [1996]; Wave III [2001–2002]; Wave IV [2008]; Wave V [2016–2018]) (15). This study used data from Waves IV, except for vegetarian status and Hispanic origin, which were from Wave III as these were not measured in Wave IV to avoid data repetition. The study and survey design has been described extensively elsewhere (15). Analyses for this study were performed using IBM SPSS Statistics, Version 25 (IBM Corp. Released 2017. IBM SPSS Statistics for Windows, Version 25.0. Armonk, NY: IBM Corp.).

### Measures

**Figure 1:**
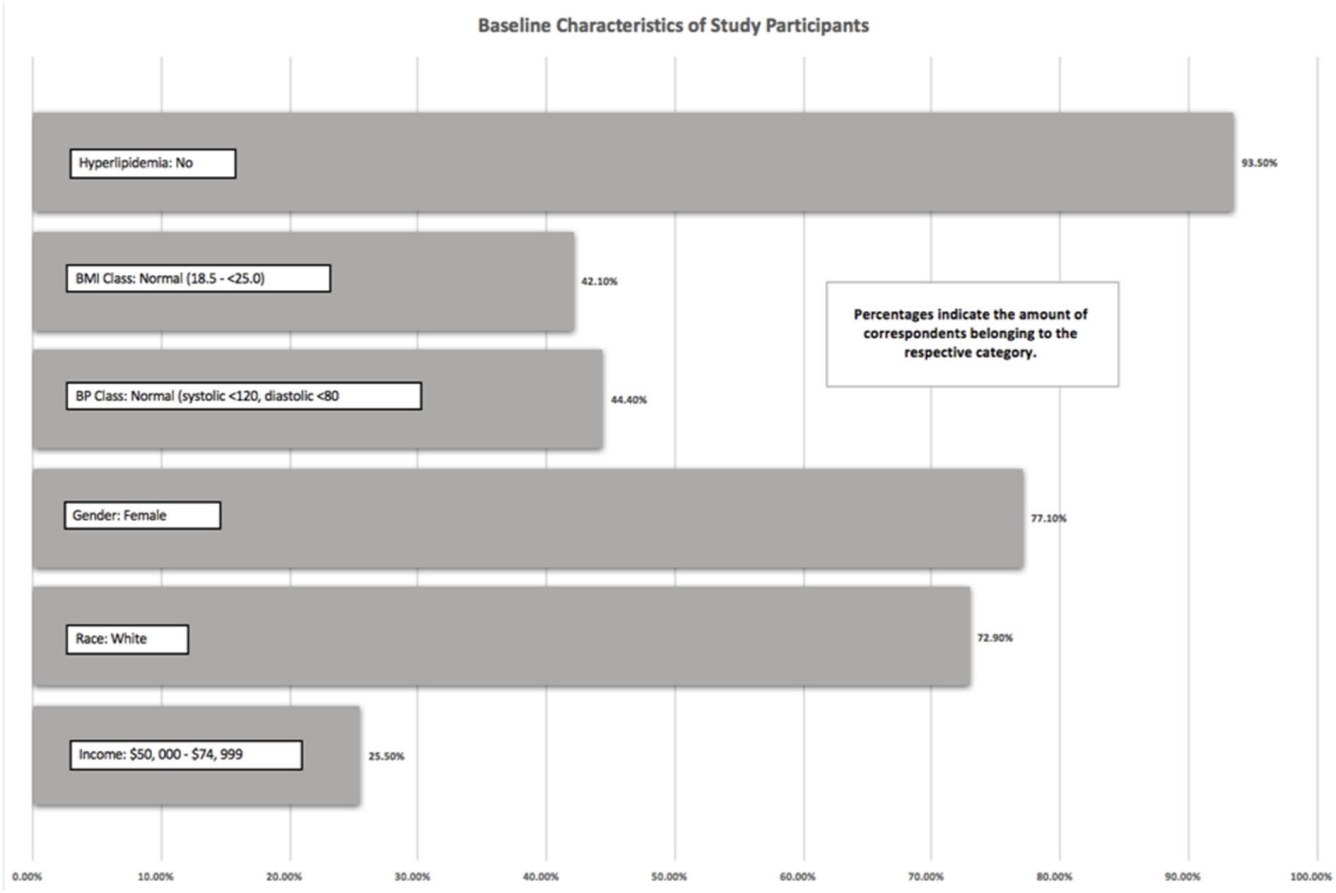
Baseline characteristics of study participants.

The Add Health study did not use any existing measures, but instead, constructed questions to meet the goals of the study (15). As previously mentioned, except for vegetarian status and Hispanic origin, which were analyzed from Wave III, all other outcomes were measured in Wave IV. The baseline descriptive statistics on the study population were reported in Figure 1 and Table 1. To assess MetS outcomes, data from Wave IV were used to investigate three components: waist circumference, systolic and diastolic blood pressure, and hyperlipidemia.

**Table 1:** Association of vegetarian status with different covariates.

**Variable**	**Number of participants**	Mode/mean^*^	**Frequency of mode**	**Percent of mode**	***p*-value**	**Odds ratio**	**95% confidence interval**
Gender	694	Women	535	77.1	0.611	0.906	0.618–1.327
Total household income	694	50–74.999 k	177	25.5	0.676	1.012	0.957–1.070
Race	694	White	506	72.9	0.462	0.928	0.762–1.131
Waist diameter (cm)^*^	694	men: 96 cm women: 92 cm	-	-	0.893	0.999	0.980–1.107
Blood pressure class	694	Normal (systolic <120, diastolic <80)	308	44.4	0.638	0.949	0.764–1.179
BMI class	694	Normal (18.5–25)	292	42.1	0.918	1.012	0.806–1.270
Hyperlipidemia	694	Normal/low	649	93.5	0.581	0.840	0.453–1.559

* *This value indicates the mean. Waist diameter is the only continuous variable. The remaining variables are categorical*.

### Vegetarian status

This was measured by asking the following question: “Do you consider yourself a vegetarian? (0 = no and 1 = yes).

### Central obesity

Interviewers measured weight, height, and waist circumference based on standardized protocols. Waist circumference was measured at the superior margin of the iliac crest for all participants that could stand unassisted, including pregnant women, to the nearest 0.5 cm. Body mass index (BMI) was also measured and classified. Detailed information can be found elsewhere (16).

### Hyperlipidemia

Interviewers asked participants if they had ever been told that they have or had high blood cholesterol or triglycerides or lipids (0 = no and 1 = yes). Due to the nature of the restricted data, we were unable to access data for the Blood Spot Assay.

### Hypertension

Certified interviewers measured participants' seated, resting systolic, and diastolic blood pressures (mmHg) and pulse rate (beats/minute) according to a standardized protocol. Three measurements were performed at 30-s intervals. Blood pressure classification was based on guidelines from the Seventh Report of the Joint National Committee on Prevention Detection, Evaluation, and Treatment of High Blood Pressure. Detailed information can be found elsewhere (16).

### Covariates

Covariates included race/ethnicity, SES, and gender. SES was measured by asking participants what the total household income was before taxes and deductions in 2006, 2007, and 2008. Participants were asked to include all sources of income, including non-legal sources. Values ranged from 1 = < $5,000 to 12= $50,000 and above.

### Data analysis

This study compared MetS components (hyperlipidemia, hypertension, and central obesity) between vegetarians and non-vegetarians. Data were used from Waves III and IV. Logistic regressions were employed to assess the association of our independent variables: hyperlipidemia, hypertension, and central obesity, along with SES, and race/ethnicity on our dichotomized dependent variable (vegetarian vs. non-vegetarian). The significance level was set at 0.05. Additionally, one-to-one propensity score matching was used to match characteristics between vegetarians and non-vegetarians with *N* = 694 matched participants (159 men and 535 women total).

## Results

### Demographic

There was a total of 694 participants in this study. As we used one-to-one matching with propensity scoring, exactly half of the participants (347 participants) were vegetarian, and half were non-vegetarian. Of the 694 participants, 93.5% of the participants did not have hyperlipidemia, 42.1% of participants had a BMI between 18.5 and 25, 44.4% of participants had blood pressure within normal limits (systolic <120 mmHg and diastolic <80 mmHg), and 25.5% had an income between $50,000 and 74,999. A total of 72.9% of the participants self-reported as white, and 77.1% of the participants identified themselves as women. These values represented the mode of each variable. The mean waist size of men was 96 cm and women was a mean of 92 cm.

### Descriptive characteristics of study participants

To determine whether a vegetarian diet was associated with the covariates (gender, total household income, race, waist diameter, blood pressure, BMI class, and hyperlipidemia), appropriate logistic regression analysis with one-to-one propensity scoring was performed. All the covariates displayed *p*-values > 0.05, indicating insignificance. Additionally, our odds ratios suggest that the presence of a vegetarian diet status was not statistically significant in its association with the covariates. Detailed values are displayed in Table 1.

## Discussion

The sample results of the current study did not find a significant association between vegetarian diet consumption in young adulthood and measures of central obesity, hypertension, and hyperlipidemia in later years. This evidence indicates a lack of direct correlation between adherence to a vegetarian diet and MetS components.

Results from previous studies indicate that the association between a vegetarian diet and traits of MetS are inconclusive, with variability seen among types of vegetarian diets. A Taiwanese cross-sectional analysis found that compared to non-vegetarians, vegetarians had significantly decreased abnormalities in BMI, systolic blood pressure, diastolic blood pressure, waist circumference, fasting blood glucose, total cholesterol, LDL, and Total cholesterol (TC): HDL ratio (OR 0.37–0.9) (13). The same study found that vegetarians exhibited greater abnormality in HDL levels (OR 1.17–1.52) and lacto-ovo vegetarians exhibited greater abnormality in Triacylglycerols (TAG) levels (OR 1.15). Unlike this cross-sectional analysis, the results of the current study showed that a vegetarian diet was not significantly associated with decreased measures of waist circumference, blood pressure, and lipid levels. This indicates that decreased BMI, BP, and TAG levels, previously thought to be associated with increased fruit and vegetable intake in a vegetarian diet compared to a non-vegetarian diet, may not be mediating MetS risk reduction among vegetarians (17). Rather, consideration of differences in the type of vegetarian diet may be more important in determining MetS risk.

On the other hand, the Taiwanese study in agreement with the current study did not find a significant difference in overall MetS risk between vegetarians and non-vegetarians but did find that a lacto-ovo vegetarian diet showed a significant difference in MetS prevalence (OR 0.87; 95% CI 0.76–0.99), under the modified ATP III criteria for Asians (13). This idea that the type of vegetarian diet may play a role in mediating MetS risk is further corroborated by a sub-study of the Adventist Health Study 2. The sub-study found that vegetarians had significantly lower levels of triglycerides, glucose, waist circumference, blood pressure, and BMI compared to non-vegetarians. Additionally, semi-vegetarians also had significantly lower BMI and waist circumference, which is one of the MetS diagnostic criteria that has also been considered in our study (17). Distinct vegetarian diets are associated with reducing certain MetS risk factors to varying degrees (5). Analyzing vegetarian diet subtypes together gives rise to inconclusiveness, potentially dependent on the distribution of the type of diet within the study population. Further investigation focused on types of vegetarian diets and MetS components is required.

Metabolic syndrome encompasses a variety of factors including dyslipidemia, glucose intolerance, hypertension, and obesity, which increase the risk for cardiovascular disease and type 2 diabetes mellitus (18). These individual factors are well-documented in literature yet, surprisingly, very little has been elucidated regarding the environmental contributions to MetS.

Attempts at elucidating the mystery behind the environment and MetS include the study done by Joseph and Vega-Lopez (19). This study looked at how an individual's perceptions of their neighborhood impacted their level of physical activity and whether it affected the prevalence of MetS in the neighborhood. This cross-sectional study sampled a population of Mexican American adults in the Phoenix, Arizona region averaging 37.9 ± 9.3 years of age. A total of 75 individuals were included in the study, 26 men and 49 women, of whom 22 individuals had MetS. In the study, slightly greater than one-third of Mexican Americans were affected by MetS, yet only around 42% achieved the national recommended physical activity levels. Data were collected using a questionnaire and were self-reported. Anthropometric assessments were also obtained, including waist size, FPG, blood pressure, serum triglycerides, and HDL cholesterol. Individuals with greater physical activity were less likely to have MetS (OR = 0.388; CI = 0.204–0.738). Regression analyses showed that factors such as a better walking environment (*R*^2^ = 0.173, B = 0.385, *p* = 0.001), a perceived safe neighborhood (*R*^2^ = 0.093, B = 0.255, *p* = 0.024), and social cohesion (*R*^2^ = 0.081, B = 0.230, *p* = 0.043) promoted physical activity. However, none of the neighborhood factors (walking environment, safety, aesthetic quality, violence, available healthy foods, social cohesion, and activities with neighbors) were found to be significantly associated with the presence of MetS, supporting physical activity as an independent risk factor for MetS, in agreement with prior studies (19).

One aspect that is not yet clear is whether an individual's genetics contribute to the development of MetS or whether an individual's environment plays a more significant role. Prior research, such as the Elder et al. (18) study compared the influence of genetics vs. environment on the development of MetS using a twin study. The study included 157 healthy adults, both men and women, ranging in age from 18 to 76 were recruited for the study. A total of 78 monozygotic twin pairs were used in the study; 29 of whom grew up in separate locations (MZA) and 49 of whom grew up in the same household (MZT). MZAs were recruited from various locations in the world whereas MZTs were primarily from the US and Canada. By comparing MZAs (genetically identical in different environments) to MZTs (genetically identical in the same environment), the researchers hoped to differentiate between genetic and environmental contributions to MetS. Intrapair correlations for body weight, BMI, waist size, and blood pressure were greater in monozygotic twins that grew up in similar environments compared to those who grew up in different environments suggesting a potential influence of the shared environment. Additionally, researchers discovered that the proportion of variance due to unique environments ranged from 13% for weight to 38% for diastolic blood pressure, while the proportion of variance due to similar environments ranged from 23% for waist circumference, to 42% for plasma glucose concentration. In each of these measurements, the proportion of variance was statistically significant. The conclusion reached in the study was that while both genetics and environment influence MetS, genetics have a predominant role in the development of MetS (18).

Some studies have taken a different approach to understanding MetS. Fernández-Rhodes et al. (20) attempted to quantify the effect of epigenetic modifications on three genes (CPT1A, SOCS3, and ABCG1) previously associated with MetS. Epigenetic modifications are heritable alterations that are not due to changes in DNA sequence but can be the result of environmental alterations. One epigenetic modification is CpG island methylation as observed in this study. Data were obtained from the Genetic Analysis Workshop (GAW20) from 188 extended families in Minnesota and Utah. These included 1,105 participants with MetS at baseline. MetS prevalence in the sample was 38.4% with a heritability of 0.47 (Standard error (SE) = 0.10; *p* = 0.00001). Fixed variables including age, age-squared, and sex, accounted for 13% of the variation in MetS. Variance components for individuals with a shared environment in early life (*c*^2^ = 0.21, SE = 0.09, *p* = 0.007) and shared environments in later life (*c*^2^ = 0.40, SE = 0.16, *p* = 0.01) were also considered. Two of the four CpG sites (one on the CPT1A gene and one on the ABCG1 gene) were highly associated with MetS (*p* = 0.00007) and another site (on the SOCS3 gene) was associated with MetS (*p* = 0.07) to a lesser extent, in the model without shared-environment related variance. Factoring in the effects of a shared environment in early life resulted in a non-significant MetS heritability estimate (*h*^2^ = 0.24, SE = 0.15, *p* = 0.05). Yet, factoring in late-life shared environments resulted in increased heritability (h^2^ = 0.46, SE = 0.12, *p* = 0.00001). The researchers concluded that while random effects in shared environments in early or late life did not drastically influence heritability, at times, the effect was significant enough to influence CpG heritability and the development of MetS. This study, like others preceding it, highlights the complex interworking of genetics and environment in the development of MetS (20).

The AddHealth data included a limited sample of buccal DNA during Wave III and DNA samples from the entire study population in Wave IV, yet did not include the CPT1A, SOCS3, and ABCG1 genes which were mentioned in the Fernandez-Rhodes study. It is hoped that DNA samples may be used in future studies to establish a link between additional genes and the development of MetS, or its individual components and whether these genes can be passed down over generations.

In conclusion, a vegetarian dietary pattern may not be as significant in reducing MetS components in later years, as has been previously thought. There are, however, several potential limitations of this study, including recall bias and dietary non-compliance. Data collection during the waves was conducted through a survey, though steps were taken during the process to reduce recall bias. Additionally, the dietary status of participants was evaluated in Wave 3 but was not re-evaluated in Wave 4. Participant dietary patterns were assumed to have remained constant across the waves. Further research is needed to better understand the role of a vegetarian diet in reducing outcomes of chronic diseases. Consideration of the variability in vegetarian diets (i.e., lacto-ovo, vegan, pescatarian, etc.) and its longitudinal association with disease outcomes are of particular interest.

## Data availability statement

Publicly available datasets were analyzed in this study. This data can be found here: AddHealth (https://addhealth.cpc.unc.edu/).

## Author contributions

RP, RA, and JC contributed to writing of the manuscript. SM conducted the statistical analysis and contributed to writing of the manuscript. ZT, HA, MY, and SH provided edits to finalize the manuscript. All authors contributed to the article and approved the submitted version.

## Conflict of interest

The authors declare that the research was conducted in the absence of any commercial or financial relationships that could be construed as a potential conflict of interest.

## Publisher's note

All claims expressed in this article are solely those of the authors and do not necessarily represent those of their affiliated organizations, or those of the publisher, the editors and the reviewers. Any product that may be evaluated in this article, or claim that may be made by its manufacturer, is not guaranteed or endorsed by the publisher.
